# Rhabdomyosarcome paratesticulaire: à propos d’un cas

**DOI:** 10.11604/pamj.2019.33.55.17269

**Published:** 2019-05-24

**Authors:** El Mahdi Graiouid, Youness Chakir, Messian Gallouo, Mohammed Dakir, Adil Debbagh, Rachid Aboutaieb

**Affiliations:** 1 Service d'Urologie, CHU Ibn Rochd, Casablanca, Maroc

**Keywords:** Paratesticulaire, rhabdomyosarcome, traitement, Paratesticular, rhabdomyosarcoma, treatment

## Abstract

Le rhabdomyosarcome (RMS) para-testiculaire est une tumeur rare. Le traitement doit être multimodal et fait appel à la chirurgie, à la chimiothérapie et à la radiothérapie. À la lumière de cette observation et d'une revue de la littérature, les auteurs discuteront les modalités diagnostiques et thérapeutiques.

## Abstract (en)

Paratesticular rhabdomyosarcoma is a rare tumor. Treatment is based on multimodal therapy as well as on surgery, chemotherapy and radiotherapy. This study and literature review highlight the diagnostic and therapeutic approaches to treat paratesticular rhabdomyosarcoma.

## Introduction

Le rhabdomyosarcome paratesticulaire est une tumeur rare et agressive. La localisation paratesticulaire est la plus fréquente des atteintes uro-génitales. Plusieurs formes sont décrites et la variante embryonnaire est la plus fréquente. Le pronostic est mauvais. La prise en charge est multidisciplinaire combinant la chirurgie, la chimiothérapie et la radiothérapie.

## Patient et observation

**Figure 1:**
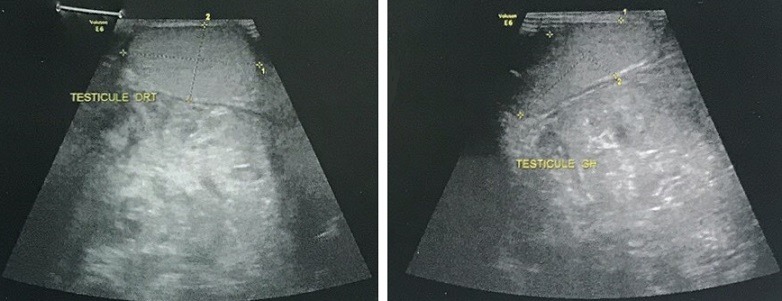
Échographie scrotale montrant la masse refoulant le testicule

M. Othmane, 19 ans, sans antécédents pathologiques particuliers consulte pour une grosse bourse indolore évoluant depuis un an et augmentant progressivement de volume. L'examen clinique trouve un patient en bon état général, apyrétique, une masse scrotale dure polylobée de 10cm de diamètre, qui semble indépendante des testicules. L'échographie réalisée à l'admission montre une volumineuse masse d'échostructure hétérogène, tissulaire, mesurant 10x7x6cm de diamètre, refoulant les deux testicules (Figure 1).

**Figure 2:**
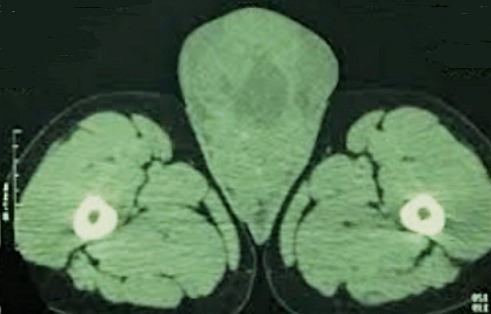
Tomodensitométrie abdominale, coupe transversale montrant la masse paratesticulaire

L'imagerie par résonance magnétique (L'IRM) montre un processus tissulaire hétérogène hyperintense en T2 à développement intrascrotale infiltrant la peau de 11x6x7cm au contact des testicules qui apparaissent refoulés sans signe d'envahissement avec présence d'adénopathies inguinales. La tomodensitométrie thoraco-abdomino-pelvienne a objectivé la présence d'une masse hétérogène intrascrotale, de plusieurs ganglions lombo-aortique gauche; ainsi que de nombreuses adénopathies inguinales bilatérales (Figure 2). Les marqueurs tumoraux étaient normaux à part une légère augmentation de LDH. Une biopsie a été réalisée objectivant un rhabdomyosarcome.

**Figure 3:**
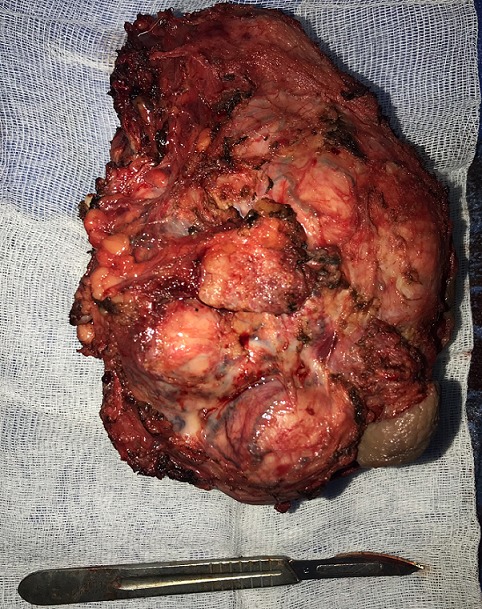
Pièce opératoire après la tumerectomie

Le patient a reçu trois cures de chimiothérapie néoadjuvante selon le protocole VAC (vincristine, doxurubicine et cyclophosphamide) sans amélioration clinique. Le volume de la masse est resté inchangé. Puis une tumerectomie par voie inguinale avec une exploration peropératoire et un envahissement des enveloppes par endroit ainsi du corps caverneux droit (Figure 3). L'examen histologique de la pièce d'orchidectomie montre un RMS embryonnaire à cellules rondes de la région paratesticulaire de 15x10x7,5cm (Figure 3). La tumeur est classée stade Ib selon la classification établie par l'Intergroup Rhabdomyosarcoma Study (IRS).

## Discussion

Les RMS paratesticulaires sont des tumeurs rares qui se voient surtout chez l'enfant et l'adolescent et représentent 7%, toute localisation confondue [1]. On admet qu'elle dérive d'éléments mésenchymateux des enveloppes du testicule, de l'épididyme ou du cordon. Le RMS paratesticulaire s'observe à tout âge mais surtout chez l'enfant et l'adulte jeune. Il y'a deux pics d'incidence, l'un à l'âge de 4 ans et l'autre à l'âge de 16 ans [2]. Il existe essentiellement trois types histologiques du rhabdomyosarcome: le type embryonnaire qui représente 97% des cas, le type alvéolaire et le type pléomorphe [3,4]. La présentation clinique n'a rien de particulier par rapport aux autres tumeurs à développement intra scrotal et le siège paratesticulaire de la tumeur est difficile à préciser par l'examen physique [5]. La découverte de la masse scrotale sera complétée par une échographie testiculaire systématique. Elle montre une masse d'échostructure hétérogène, à extension inguinoscrotale dans 80% des cas [6]. L'écho-Doppler montre un aspect hypervascularisé de la masse tumorale et précise son siège extratesticulaire aussi. La tomodensitométrie thoraco-abdomino-pelvienne permet de rechercher un envahissement des chaînes ganglionnaires profondes surtout lombo-aortiques et pelviennes et des métastases hépatiques et pulmonaires.

Macroscopiquement c'est une tumeur dont l'origine est musculaire, striée, d'aspect blanc grisâtre, de consistance ferme et encapsulée. Le diagnostic différentiel peut se poser avec les autres sarcomes paratesticulaires: léiomyosarcome, liposarcome et fibrosarcome. Il n'y a pas d'élément discriminatif entre ces différentes tumeurs, en imagerie. Leur diagnostic de certitude ne peut se faire qu'en histologie, après exérèse chirurgicale de la masse tumorale [4, 7, 8]. L'IRM est performante, utilisant des antennes de surface; la tumeur apparaît homogène, en T1 et d'aspect hétérogène, en T2 avec intensité de signal similaire au testicule normal. À cause de l'hyposignal de l'albuginée, en T2, la masse est nettement séparée du testicule [1]. Il n'y a pas de marqueurs tumoraux pouvant aider au diagnostic, qui repose uniquement sur l'examen histologique de la pièce d'orchidectomie [9]. L'analyse de la pièce d'orchidectomie et le bilan d'extension permettent la stadification de la tumeur selon l'IRS [10]. Le traitement repose sur la chirurgie, la polychimiothérapie et la radiothérapie. L'orchidectomie par voie inguinale avec ligature haute et première du cordon spermatique est le traitement standard dans les fromes localisés [11]. Le protocole de chimiothérapie le plus utilisé repose sur l'association Vincristine-actinomycine D-cycophosphamide.

## Conclusion

Le RMS paratesticulaire est une tumeur rare, qu’on retrouve surtout chez l’enfant et l’adulte jeune. Elle nécessite un diagnostic précoce et un bilan d'extension thoraco-abdomino-pelvien. Le traitement est actuellement bien codifié associant chirurgie, polychimiothérapie et radiothérapie que le pronostic s'est nettement amélioré. Une surveillance adéquate à long terme par la clinique et surtout l’imagerie, doit être instaurée afin de détecter les rechutes qui sont généralement fatales.

## Conflits d’intérêts

Les auteurs ne déclarent aucun conflit d'intérêts.
